# Effect of buserelin acetate at artificial insemination on pregnancy per AI in dairy cows inseminated with sexed semen

**DOI:** 10.29374/2527-2179.bjvm003726

**Published:** 2026-06-19

**Authors:** Mateus Gonçalves Costa, Rodrigo Vasconcelos de Oliveira, Samuel Rodrigues Bonamichi do Couto, Nicolas Moreira Piedras Monnerat Caparelli, Thiago Souza Vieira, Raphael Azevedo Lamim, Daniela Cristina Rocha de Freitas, Marco Roberto Bourg de Mello

**Affiliations:** 1 Programa de Pós-Graduação em Ciência Animal, Universidade Federal Rural do Rio de Janeiro, Seropédica, RJ, Brazil.; 2 Programa de Pós-Graduação em Medicina Veterinária, Universidade Federal Rural do Rio de Janeiro, Seropédica, RJ, Brazil.; 3 Departamento de Medicina Veterinária, Faculdade de Medicina, Universidade Federal de Juiz de Fora, Juiz de Fora, MG, Brazil.

**Keywords:** artificial insemination, dairy cattle, ovulation, pregnancy rate, pre-ovulatory follicle, inseminação artificial, bovinocultura de leite, ovulação, taxa de prenhez, folículo pré-ovulatório

## Abstract

This study determined whether administration of buserelin acetate (BA), a gonadotropin-releasing hormone agonist, during timed artificial insemination (TAI) improves pregnancy per AI (P/AI) of dairy cows inseminated with sexed semen. A total of 116 Girolando cows from three commercial dairy farms were randomly assigned to two treatment groups: Buserelin Group (BG; n = 58) that received 10 µg BA during AI, and Control Group (CG; n = 58) that did not receive an ovulation inducer. All animals were subjected to a standard ovulation-synchronization protocol, with AI performed 48 h after removing the progesterone device. Cyclicity status at the beginning of the protocol and preovulatory follicle (POF) diameter were included as covariates in statistical analysis. No significant difference in P/AI was observed between treatments (22.4% in BG vs. 13.8% in CG; *P* = 0.11). Exploratory subgroup analyses indicated a numerical tendency toward relatively high P/AI in anestrous cows treated with BA at the beginning of the protocol. Additionally, cows in BG with a POF < 13 mm showed numerically greater P/AI than did those with POF ≥ 13 mm. Overall, BA administration during AI did not significantly improve P/AI in dairy cows impregnated with sexed semen. The subgroup findings should be cautiously interpreted as the study was not specifically designed or powered for these comparisons. Further studies with relatively large populations are warranted to confirm these potential associations.

## Resumo

Este estudo determinou se a administração de acetato de buserelina (BA), um agonista do hormônio liberador de gonadotrofinas, durante a inseminação artificial em tempo fixo (IATF) melhora a taxa de prenhez por inseminação artificial (P/IA) de vacas leiteiras inseminadas com sêmen sexado. Um total de 116 vacas Girolando provenientes de três fazendas leiteiras comerciais foi aleatoriamente distribuído em dois grupos experimentais: Grupo Buserelina (GB; n = 58), que recebeu 10 µg de BA no momento da IA, e Grupo Controle (GC; n = 58), que não recebeu indutor de ovulação. Todos os animais foram submetidos a um protocolo padrão de sincronização da ovulação, com a IA realizada 48 horas após a retirada do dispositivo de progesterona. O status de ciclicidade no início do protocolo e o diâmetro do folículo pré-ovulatório (FPO) foram incluídos como covariáveis na análise estatística. Não foi observada diferença significativa na P/IA entre os tratamentos (22,4% no GB vs. 13,8% no GC; P = 0,11). Análises exploratórias de subgrupos indicaram uma tendência numérica de maior P/IA em vacas em anestro tratadas com BA no início do protocolo. Adicionalmente, vacas do GB com FPO < 13 mm apresentaram numericamente maior P/IA em comparação àquelas com FPO ≥ 13 mm. De modo geral, a administração de BA durante a IA não melhorou significativamente a P/IA de vacas leiteiras inseminadas com sêmen sexado. Os achados dos subgrupos devem ser interpretados com cautela, uma vez que o estudo não foi especificamente delineado nem teve poder estatístico para essas comparações. Estudos adicionais com populações maiores são necessários para confirmar essas potenciais associações.

## Introduction

The ability to predetermine the sex of offspring is a strategic tool to enhance the economic efficiency of cattle production systems. In dairy herds, preferential production of female calves accelerates genetic progress and optimizes replacement rates, making the use of sex-sorted semen an attractive reproductive biotechnological application. By enriching spermatozoa carrying the desired sex chromosome, this technology enables highly targeted herd expansion and improved genetic dissemination (Wellmann et al., 2024).

Despite these advantages, the use of sexed semen remains associated with reduced fertility compared to that of conventional semen (Mallory et al., 2013; Pirez et al., 2020). Spermatozoa experience mechanical stress, laser exposure, dilution, and cryopreservation during flow cytometric sorting, which collectively compromise membrane integrity, mitochondrial function, and overall viability (Yotov et al., 2023). Additionally, sex-sorted sperm exhibits reduced longevity within the female reproductive tract and may undergo capacitation earlier than conventional sperm, thereby narrowing the optimal fertilization window (Lu & Seidel Junior, 2004). Consequently, precise synchronization between ovulation and insemination becomes a critical determinant of pregnancy-related success when using sexed semen (Seidel Junior & DeJarnette, 2022). Even minor deviations in temporal relationship between sperm availability and oocyte release may significantly impair conception rates, underscoring the sensitivity of sex-sorted spermatozoa to the timing of ovulation (Kasimanickam et al., 2025).

In cattle subjected to ovulation-synchronization protocols, administration of gonadotropin-releasing hormone (GnRH) analogues during artificial insemination (AI) increases pregnancy per artificial insemination (P/AI), particularly in animals that fail to express estrus (Cedeño et al., 2021). GnRH-induced stimulation of the anterior pituitary promotes a synchronized and amplified surge of luteinizing hormone (LH), thereby enhancing ovulatory response and reducing variability in ovulation timing (Berean et al., 2023; Burnett et al., 2022). By ensuring a relatively highly predictable interval between insemination and ovulation, GnRH analogues may improve sperm–oocyte synchrony, a factor of particular relevance when sperm lifespan is limited.

Although the beneficial effects of GnRH administration at AI have been documented in programs using conventional semen, evidence regarding its efficacy in cows impregnated with sexed semen remains limited. Reduced durability and heightened susceptibility of sex-sorted spermatozoa to temporal mismatches highlight the need to evaluate whether hormonal induction of ovulation can mitigate these physiological constraints. Furthermore, limited studies have explored the interaction between GnRH administration and intrinsic reproductive variables, such as cyclicity status and preovulatory follicle (POF) diameter, which influence ovulatory competence and subsequent luteal function. This gap in knowledge supports further investigation into endocrine strategies aimed at optimizing conception outcomes in programs involving sexed semen.

Buserelin acetate (BA) is a synthetic GnRH analogue, which is widely used in reproductive management to induce ovulation and enhance luteal function (Pandey et al., 2015; Thatcher et al., 2003). By stimulating the release of LH and follicle-stimulating hormone (FSH) from the pituitary gland, BA promotes ovulatory response and may reduce dispersion in ovulation timing. In cattle, BA administration increases ovulation rates and improves reproductive performance under specific conditions (Silva et al., 2024). Given the constrained lifespan of sexed spermatozoa, the use of BA during AI may enhance the success of fertilization by improving ovulatory synchrony and ensuring highly precise temporal overlap between viable sperm and the oocyte.

Therefore, we hypothesize that BA administration during AI increases P/AI in dairy cows impregnated with sexed semen. Specifically, the objectives were to compare P/AI between cows receiving or not receiving BA during AI and to evaluate the influence of cyclicity status and POF diameter on pregnancy outcomes in cows impregnated with sexed semen.

## Material and methods

### Experimental period and ethical approval

The experiment was conducted in January and February of 2022 and 2023. All study procedures were conducted in accordance with ethical principles of animal experimentation and approved by the Animal Use Ethics Committee of Institute de Zootecnia at the Universidade Federal Rural do Rio de Janeiro (CEUA–IZ–UFRRJ) under process number 0200-07-2023.

### Animals, inclusion criteria, and management

A total of 116 multiparous Girolando females (*Bos taurus* × *Bos indicus*), non-pregnant and clinically healthy with respect to uterine and ovarian pathologies, were enrolled in this study. The inclusion criteria were a minimum of 60 days in milk, body condition score (BCS) ≥ 3.0 (on a 1–5 scale, in which 1 = emaciated and 5 = obese), and absence of reproductive abnormalities at the initial ultrasonographic examination. Cows had an average milk yield of approximately 25 L/day, obtained through daily milking twice. Animals were maintained under a semi-confinement system and received a diet containing commercial concentrate, mineral supplementation, and corn silage provided in the feed bunk. Grazing areas predominantly contained buffel grass (*Cenchrus ciliaris*), and water was provided *ad libitum*.

### Ultrasonographic evaluations

Transrectal ultrasonographic examinations were performed using a DP-10 Vet Power ultrasound machine (Mindray; equipped with a 7.5-MHz linear transducer) at three experimental time points. At the beginning of the synchronization protocol, ovarian status was assessed; females presenting a corpus luteum (CL) in either ovary were classified as cyclic, whereas those without a detectable CL were classified as anestrous. On the day of timed artificial insemination (TAI), POF diameter was measured in B-mode, and follicular diameter was calculated as mean of the two largest perpendicular transverse measurements. Pregnancy was diagnosed by transrectal ultrasonography between 30 and 40 days after TAI and confirmed by visualization of an embryonic vesicle containing a viable embryo with detectable heartbeat.

### Ovulation-synchronization protocol

The ovulation-synchronization protocol was initiated on a random day of the estrous cycle (designated day 0, D0) with the insertion of an intravaginal progesterone device (Primer Monodose; Agener União Saúde Animal) combined with intramuscular administration of 2 mg estradiol benzoate (Gonadiol; Zoetis). On D9, the progesterone device was removed, and cows received intramuscular injections of 300 IU equine chorionic gonadotropin (eCG; Ecegon; Biogenesis Bagó), 500 µg cloprostenol sodium (Estron; Agener União Saúde Animal), and 1 mg estradiol cypionate (Fertilcare Ovulação; MSD Saúde Animal Brasil). TAI was performed 48 h after removing the progesterone device, and only the first insemination service was included in subsequent analysis.

### Experimental design and treatments

During TAI, females were randomly allocated into two experimental groups. The Buserelin Group (BG; n = 58) was intramuscularly administered 10 µg BA (Sincroforte; Ourofino Saúde Animal) simultaneously during TAI, whereas the Control Group (CG; n = 58) was impregnated with sexed semen without the administration of a GnRH agonist. A single experienced technician performed all inseminations using sexed semen (Sexecel; ABS Global Brasil) from eight different bulls, and the proportion of cows inseminated per bull was maintained similar across treatment groups. To control for potential sire fertility effects, the variable “bull” was included in the statistical model.

### Statistical analysis

Pregnancy rate (pregnant/non-pregnant) was analyzed by multivariate logistic regression using BioEstat v.5.3 by including treatment (BG vs. CG), ovarian cyclicity at D0 (cyclic vs. anestrous), BCS, animal category, farm, bull, POF diameter, and biologically plausible interactions as explanatory variables. All variables were initially included in the model, and nonsignificant effects were sequentially removed based on the Wald test criterion (*P* > 0.20). Statistical significance was set at *P* < 0.05, whereas values between 0.05–0.10 were considered trends. POF diameter, a continuous variable, was evaluated for normality and homogeneity of variances prior to mean comparisons by analysis of variance (ANOVA). For complementary analysis, POF diameter was categorized according to the overall mean (13 mm) into two groups (POF < or ≥ 13 mm). P/AI between treatments were also compared using the binomial test for two independent proportions, adopting the normal approximation criteria for binomial distribution (n_1_p_1_q_1_ ≥ 5 and n_2_p_2_q_2_ ≥ 5). Continuous data are presented as mean ± standard error (SE), and categorical outcomes as percentages.

## Results

The mean diameter of POFs at the time of TAI, distribution of ovarian status at the beginning of synchronization, and overall pregnancy outcomes are presented in Table 1. Groups were homogeneous regarding POF diameter and ovarian status at protocol initiation. Although P/AI was numerically higher in BG than in CG, no significant effect of treatment was observed (*P* = 0.11).

**Table 1:** Mean diameter of preovulatory follicle (POF) on the day of insemination (± standard error [SE]), percentage of anestrous and cyclic cows at the start of the synchronization protocol, and pregnancy per insemination (P/AI) of crossbred dairy females inseminated at fixed time with sexed semen with or without administration of buserelin acetate.

**Parameters**	**Groups**		***P-*value**
	**BG**	**CG**	
Mean diameter POF (mm)	13.1 ± 0.06	12.9 ± 0.06	0.84
Anestrus (%)	62.0 (36/58)	70.7 (41/58)	
Cyclic (%)	38.0 (22/58)	29.3 (17/58)	
P/AI (%)	22.4 (13/58)	12.1 (7/58)	0.11

BG: buserelin acetate group; CG: control group.

The association between ovarian status at the beginning of synchronization and P/AI is presented in Table 2. No association between ovarian status and P/AI was observed within treatments. However, anestrous cows in BG showed a numerical tendency toward greater P/AI compared with those in CG (*P* = 0.07), whereas P/AI was similar between treatments in cyclic cows (*P* = 0.48).

**Table 2:** Effect of ovarian condition (anestrus or cyclic) of dairy cows at the start of the synchronization protocol on pregnancy per insemination (P/AI) in different treatment groups.

**Ovarian condition**	**P/AI (%)**		***P-*value**
	**BG**	**CG**	
Anestrus	25.0 (9/36)	12.2 (5/41)	0.07
Cyclic	18.2 (4/22)	17.6 (3/17)	0.48
*P-*value	0.29	0.27	

BG: buserelin acetate group; CG: control group.

The overall mean POF diameter at TAI was 13.0 ± 0.06 mm. The association between POF category and P/AI is presented in Table 3. In BG, cows with POF < 13 mm had P/AI higher than that of cows with POF ≥ 13 mm (*P* = 0.01). In CG, a similar tendency was observed (*P* = 0.08). No differences between treatments were observed within each POF category.

**Table 3:** Effect of preovulatory follicle (POF) diameter in dairy cows during timed artificial insemination (TAI) on pregnancy per insemination (P/AI).

**Preovulatory follicle diameter on the day of TAI**	**P/AI (%)**		***P-*value**
	**BG**	**CG**	
POF ≥ 13 mm	6.7	5.9	0.46
POF < 13 mm	39.1	21.7	0.10
*P-*value	0.01	0.08	

BG: buserelin acetate group; CG: control group.

## Discussion

The present study evaluated the effect of BA administration during TAI on P/AI of Girolando crossbred dairy females inseminated with sexed semen. The initial hypothesis that BA would increase P/AI was not confirmed because no significant difference was observed between BG and CG. Although exploratory subgroup analyses identified differences according to ovarian status and follicular diameter, these findings should be cautiously interpreted.

Previous studies evaluating the effects of administering GnRH analogues at the time of insemination have mainly focused on protocols using conventional semen (Berean et al., 2023; Burnett et al., 2022). In these systems, GnRH-induced synchronization of ovulation may improve fertility by enhancing ovulatory synchrony and preovulatory LH surge (Burnett et al., 2022). Chenault (1990) has reported improved pregnancy rates following BA administration in cattle inseminated with conventional semen. However, sexed semen substantially differs from conventional semen because of its relatively low sperm concentration and short functional lifespan within the female reproductive tract (Frijters et al., 2009). Therefore, small variations in ovulation timing may have a high impact on sperm–oocyte synchrony, potentially contributing to the absence of an overall treatment effect observed in the present study.

Although no overall treatment effect was observed, anestrous cows receiving BA showed a numerical tendency toward greater P/AI. Ovarian status at the beginning of synchronization protocols is associated with reproductive performance (Bisinotto et al., 2010), and cyclic cows generally experience greater progesterone exposure during follicular development, which favors oocyte competence and fertility (Wiltbank et al., 2014). In contrast, follicular development under low progesterone concentrations, commonly observed in anestrous cows, may compromise oocyte quality (Denicol et al., 2012). Therefore, in the present study, BA administration at TAI might have partially improved ovulatory synchrony in anestrous cows. Nevertheless, this finding should be cautiously interpreted, particularly because eCG was administered at progesterone-device removal, which might have reduced physiological differences between cyclic and anestrous cows (Bryan et al., 2013; Shephard, 2013).

An association between POF diameter and P/AI was also observed in the present study. Follicle size at TAI is associated with ovulation rate, development of corpus luteum, and progesterone production during early diestrus (Bonato et al., 2022; Morotti et al., 2022; Yotov et al., 2023). However, inconsistent associations have been reported between follicular diameter and fertility outcomes, particularly under field conditions (Yotov et al., 2023). In the present study, cows with POF < 13 mm in BG showed relatively high P/AI, and a similar tendency was observed in CG. These results suggest that the relationship between follicular diameter and fertility may be independent of BA treatment and should not be interpreted as evidence of a specific treatment effect in cows with relatively small follicles. Probably, relatively small follicles at TAI might still be physiologically competent and ovulate within an adequate time window relative to insemination. Conversely, relatively large follicles might have represented relatively advanced or persistent follicles, which were associated with altered endocrine conditions and reduced oocyte competence (Mihm et al., 1999; Roth et al., 2012). Nevertheless, because ovulation timing and endocrine responses were not evaluated, these interpretations remain speculative.

This study has some limitations. Circulating concentrations of LH and progesterone were not measured, and the precise timing of ovulation relative to insemination was not determined. Therefore, mechanistic inferences regarding endocrine responses to BA remain speculative. Additionally, subgroup analyses were exploratory in nature, and the study was not specifically powered to detect differences within these categories.

## Conclusion

BA administration during TAI did not increase overall pregnancy per AI in Girolando dairy females inseminated with sexed semen. The absence of an overall treatment effect and exploratory nature of subgroup analyses prevent definitive conclusions regarding specific categories of animals. Further studies evaluating endocrine dynamics, ovulation timing, and relatively large populations are warranted to clarify potential interactions observed in the present study.
